# Neonatal respiratory distress syndrome revealing a cervical bronchogenic cyst: a case report

**DOI:** 10.1186/s12887-015-0363-2

**Published:** 2015-06-27

**Authors:** Penelope Thaller, Catherine Blanchet, Maliha Badr, Renaud Mesnage, Nicolas Leboucq, Michel Mondain, Gilles Cambonie

**Affiliations:** Department of Neonatology and Pediatric Intensive Care Unit, Hôpital Arnaud de Villeneuve, 371 Avenue du Doyen Gaston Giraud, 34295 Montpellier Cedex 5, France; Department of Pediatric Otorhinolaryngology, Hôpital Arnaud de Villeneuve, 371 Avenue du Doyen Gaston Giraud, 34295 Montpellier Cedex 5, France; Department of Neuroradiology, CHU Montpellier, F-34000 Montpellier, France

**Keywords:** Cervical bronchogenic cyst, Neonatal respiratory distress syndrome, Tracheostomy

## Abstract

**Background:**

Bronchogenic cyst is a congenital malformation, rarely located in the cervical region and almost never involved in a neonate with acute respiratory distress in the delivery room.

**Case presentation:**

A female newborn with respiratory distress syndrome caused by a large left cervical mass. Intubation was difficult due to tracheal deviation. Magnetic resonance imaging confirmed a left cervical cyst displacing the trachea and esophagus laterally. Surgical excision was performed via a cervical approach on the 5th day, and pathological examination revealed a bronchogenic cyst. The patient's course was complicated by left vocal cord paralysis and necrotic lesions in the glottic and subglottic regions; she required a tracheostomy on the 13th day. Inflammatory stenosis in the subglottic region required balloon dilation once, 20 days later. Proximal esophageal stenosis induced transient upper airway obstruction with salivary stasis. Decannulation was performed at 2 months and the patient was discharged 10 days later.

**Conclusion:**

A bronchogenic cyst can exceptionally obstruct the airways in the neonatal period. Surgical excision is necessary, but postoperative complications may occur if the cyst is in close contact with the trachea and esophagus, including necrotic and stenotic lesions of the upper aerodigestive tract. In those situations, tracheostomy may be necessary for mechanical ventilation weaning and the initiation of oral feeding.

## Background

Bronchogenic cyst is a congenital malformation of the tracheobronchial tree. It is usually located in the mediastinum or lung parenchyma, though rarely can be seen in extrathoracic locations such as the neck. In 2004, Mehta et al. reported a series of 4 cases of cervical bronchogenic cysts over a 22-year period, with the age of discovery ranging from 3 weeks to 6 years [1]. In 2008, Teissier et al. reported 8 cases over a period of 13 years [2]. The signs and symptoms are variable, but usually consistent with a neck mass associated with chronic cough or stridor [3]. Some cases are not diagnosed until adulthood, when an isolated laterocervical mass is discovered incidentally [4]. We report an exceptional case of cervical bronchogenic cyst revealed by acute respiratory distress in the neonatal period.

## Case presentation

The patient’s mother received prenatal care during pregnancy, with 3 antenatal US exams showing normal fetal development. Labor was induced at 41^+4^ gestational weeks in a type 2a maternity ward and delivery was by cesarean section because of abnormal fetal heart rate. The newborn was female with a birth weight of 3045 g. The newborn showed ineffective ventilation from the first postnatal minute, and positive pressure ventilation with a bag and mask was immediately started. A left cervical swelling was also observed. Respiratory distress, with suprasternal tugging and stridor, occurred at every attempt to withdraw ventilatory support. Furthermore, FiO_2_ of 90 % was required to maintain SpO_2_ above 90 %. Two attempts of orotracheal intubation failed because the larynx could not be visualized. A more experienced pediatrician was called for help. Standard direct laryngoscopy found a right laryngeal deviation with a restricted laryngeal orifice. After sedation with midazolam (0.1 mg/kg intravenous, 2 doses 10 min apart), tracheal intubation was achieved at the 3rd attempt, with a 2.5-mm endotracheal tube, at 1 h 30 min after birth. The newborn was then transferred to a type 3 neonatal unit.

**Fig. 1:**
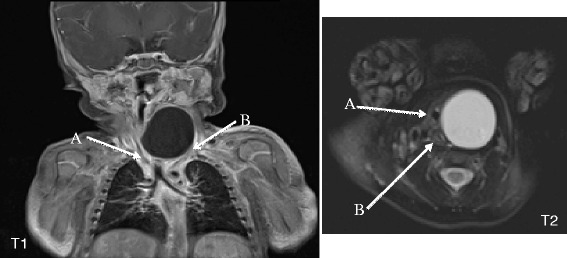
Cervical thoracic MRI. Coronal T1-weighted sequence showing a voluminous left laterocervical mass displacing the trachea (**a**) and carotid-jugular axis (**b**). Axial T2-weighted sequence showing tracheal compression (**a**) and esophageal compression (**b**) by the cyst

On admission, the newborn was ventilated in assist-control pressure mode with FiO_2_ set at 21 %. Clinical examination confirmed a noninflammatory cervical swelling that was soft on palpation. Chest x-ray revealed the endotracheal tube deflected to the right with respect to the spinal axis. Cervical US showed a thin-walled cystic mass measuring 24 × 28 × 37 mm displacing the trachea and the left thyroid lobe medially and forward, and the carotid and jugular vessels laterally and posteriorly. MRI identified a large cystic mass with well-defined walls that laterally displaced the aerodigestive tract (Fig. 1).

In the first 48 h of management, peak inspiratory pressures > 25 cm H_2_O were necessary to observe chest expansion and hear vesicular breath sounds at auscultation. Volume mode was tested, but it also generated high inspiratory pressures, between 25 and 30 cm H_2_O, despite the selection of a minimal tidal volume (5 ml/kg) and the use of permissive hypercapnia (PCO_2_ allowed to rise as high as 60 mmHg). On the assumption that the cyst might be squeezing the endotracheal tube, we performed fine needle aspiration of the cyst in the operating room, under general anesthesia, on postnatal day 3. Twenty milliliters of clear liquid was removed for cytological analysis, which revealed both respiratory-type epithelium and squamous epithelium. Rigid bronchoscopy performed at the same time confirmed tracheal compression and no laryngeal fistula. A 3-mm endotracheal tube was put into place to secure the airway. On day 5, surgery using a cervical approach isolated a unilocular cystic structure measuring 25 × 23 × 6 mm in close contact with the trachea and esophagus. Dissection was not possible from the trachea or the esophagus, and limited resection of trachea and esophagus was therefore mandatory, resulting in an opening of both structures. Both were then sutured. Histological examination showed that the cyst wall was composed of fibrous and smooth fibromuscular tissue, with some seromucinous bronchial gland cells. The wall was lined by a layer of respiratory-type ciliated cells. These findings suggested the diagnosis of bronchogenic cyst.

**Fig. 2:**
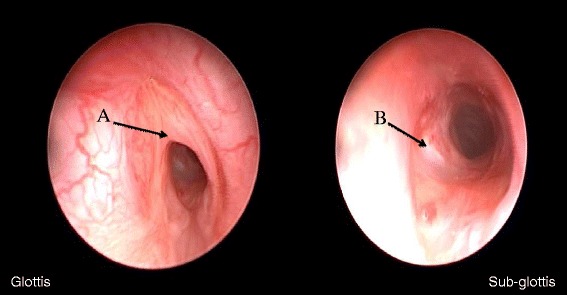
Postoperative endoscopy. Glottic region: anterior synechia (**a**) causing luminal stenosis of 50 %. Subglottic region: sutured tracheal wound (**b**) and inflammatory alterations causing grade 1 stenosis

After the first attempt at extubation on the day after surgery, the infant had respiratory distress due to laryngotracheal edema and left vocal fold paresis that was refractory to treatment with nebulized epinephrine and corticosteroids and required reintubation. The second attempt at 8 days postsurgery was equally unsuccessful. The upper respiratory tract was explored under general anesthesia at a postnatal age of 13 days. We observed edema of the left laryngeal ventricle and the anterior third of the glottis, associated with the necrotic appearance of the mucosa in the left lateral subglottis (Fig. 2). Such lesions in a newborn who could not be weaned from mechanical ventilatory support prompted immediate tracheostomy.

Sedation was gradually reduced over 48 h postsurgery, and mechanical ventilation was ended 2 weeks later with the infant ventilating effectively with the tracheal cannula.

Laryngotracheobronchoscopy under general anesthesia on day 33 revealed a subglottic stenosis treated with a 6-mm diameter balloon dilation. Proximal esophageal stenosis was also observed and not treated. Videographic exploration of swallowing confirmed the images of a very proximal partial esophageal stenosis, from C3 to C4.

After a final endoscopic assessment at 2 months showing the absence of airway stenosis*,* the cannula was withdrawn. The infant was then discharged 10 days later. She was exclusively breastfed and weight and height gains were satisfactory, with respective gains of 915 g and 7 cm since birth, with absence of swallowing difficulty in relation to the partial esophageal stenosis.

## Discussion

The respiratory bud develops from the ventral side of the primitive foregut from the 4th week of development. Cervical bronchogenic cyst may result from an abnormal division of the bud and migration to an ectopic localization in the neck region [1,5,6]. Other extrathoracic localizations have been described, including subcutaneous [7] and even abdominal [8] sites.

Antenatal discovery of a bronchogenic cyst is rare and localization is generally intrathoracic [9,10]. One case has been reported of a cervico-mediastinal bronchogenic cyst detected by a cervical mass on prenatal US [11]. Undetected cervical bronchogenic cyst may also cause polyhydramnios by esophageal compression [12]. In both this case and ours, an abnormal fetal heart rate precipitated delivery by cesarean section. Nevertheless, the direct and indirect impact of this malformation on disturbances in fetal monitoring is difficult to establish.

The immediate neonatal respiratory distress of our patient was explained by direct compression of the trachea, causing major difficulties for intubation because the larynx could not be visualized. The use of a video-laryngoscope for intubation can improve the viewing conditions of the upper airways, making it easier to identify the laryngeal inlet. Rigid bronchoscopy or a flexometallic tube can also relieve glottic-subglottic compression, although great caution is required while advancing the scope or the tube. The ex utero intrapartum treatment (EXIT) procedure can also be done if prenatal diagnosis suggests fetal airway obstruction by a neck mass. Its use in this context has mainly been reported for cervical lymphatic malformations and cervical teratomas [13,14].

The main differential diagnosis is cystic hygroma, which is more likely in the setting of a multilocular cyst, and more unlikely in the setting of a unilocular cyst. Other differential diagnoses include thymic cyst, branchial cyst and thyroglossal duct cyst, but they are rarely responsible for neonatal respiratory distress. Only histological examination of the resected specimen confirms the diagnosis.

MRI is essential before surgery, as it provides the surgeon with precise information on the anatomical relationships with adjacent organs [15]. Rigid bronchoscopy assesses the degree of cyst-trachea contact and the need for tracheal surgery [12]. Needle aspiration of the cyst usually has low diagnostic value [16]. It was performed in our patient to temporarily reduce the tracheal compression. However, neither cyst aspiration nor the larger caliber endotracheal tube introduced following bronchoscopy significantly reduced peak inspiratory pressures, which remained high until surgery.

The only treatment is surgical excision, even for an asymptomatic cyst, given the risks of infection, hemorrhage and, rarely, malignant transformation [17]. The paralysis of the left vocal cord may have resulted from direct injury to the laryngeal nerve during surgery. Paralysis was reported in two other cases [2,12], but in one of these cases this condition had already been observed during the preoperative bronchoscopy, suggesting direct mechanical interference between the cyst and the ipsilateral laryngeal nerve [2]. Evaluation of vocal cord mobility before surgery was not possible in our case because of the depth of sedation during bronchoscopy.

Several factors probably contributed to the alterations in the glottic and subglottic regions observed after the intervention, including intubation and the repeated attempts to intubate, the inflammation associated with laryngeal surgery, and the prolonged mechanical ventilation. In a newborn, edemic and necrotic lesions further narrow an already physiologically narrow larynx, which is an additional motivation to perform tracheostomy.

In addition to the vocal cord paralysis, persistent airway obstruction after laryngeal surgery should always raise the suspicion of a local cause, such as stenosis. In our patient, a proximal esophageal stenosis was also present after surgery. The damage to the esophageal mucosa was probably caused by the surgery, secondary to cyst resection and the suturing of borders. This stenosis, however, had only transient and moderate clinical consequences and did not preclude satisfactory breastfeeding, even with the tracheal cannula.

## Conclusion

Cervical bronchogenic cyst is rare and diagnosis is most often made in childhood. However, it should be systematically considered in all cases of neonatal cervical tumor, especially if respiratory distress is present. Prenatal diagnosis may be guided by an unexplained polyhydramnios and/or direct visualization of a cyst, in which case delivery should be performed in a maternity hospital with a type 3 neonatal unit. The EXIT procedure, with a multidisciplinary team approach, should also be planned for fetuses with evidence of airway obstruction on prenatal images.

## Consent

Written informed consent was obtained from the parents of the patient for publication of this case report and the accompanying images. A copy of the written consent is available for review by the Editor of this journal.

## Abbreviations

US: Ultrasound
FiO_2_: Fraction of inspired oxygen
SpO_2_: Oxygen saturation by pulse oximetry
MRI: Magnetic resonance imaging
cm H_2_O: centimeter of water (0.7355 mmHg)
PCO_2_: Partial pressure of carbon dioxide measured on capillary blood gas sampling (mmHg)
C3: 3rd cervical vertebra
C4: 4th cervical vertebra
